# Studying concatenation of the Cas9-cleaved transgenes using barcodes

**DOI:** 10.18699/vjgb-25-04

**Published:** 2025-02

**Authors:** A.V. Smirnov, A.N. Korablev, I.A. Serova, A.M. Yunusova, A.A. Muravyova, E.S. Valeev, N.R. Battulin

**Affiliations:** Institute of Cytology and Genetics of the Siberian Branch of the Russian Academy of Sciences, Novosibirsk, Russia; Institute of Cytology and Genetics of the Siberian Branch of the Russian Academy of Sciences, Novosibirsk, Russia; Institute of Cytology and Genetics of the Siberian Branch of the Russian Academy of Sciences, Novosibirsk, Russia; Institute of Cytology and Genetics of the Siberian Branch of the Russian Academy of Sciences, Novosibirsk, Russia; Novosibirsk State University, Novosibirsk, Russia; Institute of Cytology and Genetics of the Siberian Branch of the Russian Academy of Sciences, Novosibirsk, Russia; Institute of Cytology and Genetics of the Siberian Branch of the Russian Academy of Sciences, Novosibirsk, Russia Novosibirsk State University, Novosibirsk, Russia

**Keywords:** CRISPR/Cas9, pronuclear microinjection, DNA barcoding, transgenic animals, DSB repair, concatemer, homologous recombination (HR), non-homologous end-joining, NHEJ, mouse embryos, CRISPR/Cas9, пронуклеарная микроинъекция, ДНК-баркодирование, трансгенные животные, репарация двуцепочечных разрывов ДНК, конкатемер, гомологичная рекомбинация, негомологичное соединение концов, эмбрионы мыши

## Abstract

In pronuclear microinjection, the Cas9 endonuclease is employed to introduce in vivo DNA double-strand breaks at the genomic target locus or within the donor vector, thereby enhancing transgene integration. The manner by which Cas9 interacts with DNA repair factors during transgene end processing and integration is a topic of considerable interest and debate. In a previous study, we developed a barcode-based genetic system for the analysis of transgene recombination following pronuclear microinjection in mice. In this approach, the plasmid library is linearized with a restriction enzyme or a Cas9 RNP complex at the site between a pair of barcodes. A pool of barcoded molecules is injected into the pronucleus, resulting in the generation of multicopy concatemers. In the present report, we compared the effects of in vivo Cas9 cleavage (RNP+ experiment) and in vitro production of Cas9- linearized transgenes (RNP– experiment) on concatenation. In the RNP+ experiment, two transgenic single-copy embryos were identified. In the RNP– experiment, six positive embryos were identified, four of which exhibited lowcopy concatemers. Next-generation sequencing (NGS) analysis of the barcodes revealed that 53 % of the barcoded ends had switched their initial library pairs, indicating the involvement of the homologous recombination pathway. Out of the 20 transgene-transgene junctions examined, 11 exhibited no mutations and were presumably generated through re-ligation of Cas9-induced blunt ends. The majority of mutated junctions harbored asymmetrical deletions of 2–4 nucleotides, which were attributed to Cas9 end trimming. These findings suggest that Cas9-bound DNA may present obstacles to concatenation. Conversely, clean DNA ends were observed to be joined in a manner similar to restriction-digested ends, albeit with distinctive asymmetry. Future experiments utilizing in vivo CRISPR/ Cas cleavage will facilitate a deeper understanding of how CRISPR-endonucleases influence DNA repair processes.

## Abstract (ru)

При пронуклеарной микроинъекции эндонуклеаза Cas9 используется для создания двуцепочечных разрывов ДНК in vivo в геномном целевом локусе или внутри донорского вектора для улучшения интеграции трансгена. Взаимодействие Cas9 с факторами репарации ДНК во время обработки концов трансгена и интеграции – актуальная тема. Ранее мы разработали генетическую систему на основе баркодов для анализа рекомбинации трансгенов после пронуклеарной микроинъекции у мышей. При этом подходе плазмидная библиотека линеаризуется ферментом рестрикции или комплексом Cas9:gRNA на участке между парой баркодов. Пул баркодированных молекул вводится внутрь пронуклеуса, что приводит к образованию многокопийных конкатемеров. В представленной статье мы сравнили эффекты разрезания Cas9 в условиях in vivo (эксперимент RNP+) или для трансгенов, линеаризованных in vitro и очищенных через агарозный гель (эксперимент RNP–). В эксперименте RNP+ было обнаружено два трансгенных однокопийных эмбриона. В эксперименте RNP– было идентифицировано шесть положительных эмбрионов, четыре из них имели низкокопий- ные конкатемеры. Анализ баркодов методом NGS показал, что 53 % баркодированных концов поменяли свои исходные пары баркодов, что является признаком гомологичной рекомбинации между концами трансгенов. Из 20 слияний «трансген-трансген» 11 не имели мутаций и, по-видимому, были получены путем повторного лигирования тупых концов, полученных с помощью Cas9. Большинство мутировавших соединений содержало асимметричные делеции 2–4 нуклеотидов из-за специфического тримминга концов Cas9. Эти данные указывают на то, что связанная с Cas9 ДНК создает препятствия для конкатенации. В то же время свободные концы ДНК соединяются таким же образом, как и концы, обработанные рестриктазами, но с характерной асимметрией. Будущие эксперименты с пронуклеарными микроинъекциями CRISPR/Cas помогут понять, как CRISPR-нуклеазы влияют на репарацию ДНК.

## Introduction

Pronuclear microinjection is a common method for making transgenic animals. At present, CRISPR/Cas nucleases are successfully employed in pronuclear microinjections to produce site-specific knock-ins and mutations. The introduction of site-specific double-strand breaks (DSBs) has been demonstrated to enhance the frequency of editing when singlestranded oligonucleotides or long double-stranded donors are incorporated. Moreover, Cas9 can be utilized to cleave circular donor vectors in vivo, thereby providing doublestranded ends for repair (Abe et al., 2020). Approaches such as minicircles (Danner et al., 2021) or HITI/PiTCh (Sakuma et al., 2016; Suzuki et al., 2016) are based on non-homologous or microhomology-mediated end-joining (NHEJ, MMEJ) and have been reported to have a high editing frequency due to the fact that NHEJ/MMEJ is active throughout the cell cycle. In contrast to the predominantly utilized clean ends in classical transgenesis, Cas9-cleaved DNA engages with the cellular DNA repair apparatus in a distinctive manner. For example, the binding of the Cas9 complex to the target site results in the local denaturation of the DNA duplex and trimming of the cleaved ends, which then form single-stranded overhangs of varying lengths (Stephenson et al., 2018). Following DNA cleavage, Cas9 remains bound to the cut site for several hours, tethering the two ends together (Richardson et al., 2016). This prevents immediate end processing by DNA repair factors and necessitates the removal of Cas9 by cell factors to seal the break (Clarke et al., 2018; Reginato et al., 2024).

The majority of our knowledge regarding the Cas9 mode of action is derived from cell culture assays utilizing site-specific reporters (Schimmel et al., 2017) and next-generation indel sequencing (NGS) (Schimmel et al., 2017; Taheri-Ghahfarokhi et al., 2018). We elected to investigate the manner in which Cas9-cleaved ends engage with DSB repair systems, employing a novel assay based on molecular barcoding and concatenation in pronuclear microinjection. The concatenation of the injected transgenes into multicopy arrays is a well-documented aspect of transgenesis, occurring with high frequency in pronuclear microinjection. Molecular barcode evidence indicates that concatenation is facilitated by the combined action of homologous recombination (HR) (head-to-tail recombination) and NHEJ (random ligation and initial copy circularization) (Smirnov et al., 2020). During the process of concatenation, the repair pathways leave specific signatures at the junctions between the transgenes and in the patterns of connection between the barcode and the transgenes. Therefore, the levels of barcode recombination and terminal truncations may serve as indicators of the accessibility of DNA ends to DNA repair machinery in the presence of the Cas9 nuclease.

To ascertain the impact of Cas9-generated ends on concatenation, a barcoded plasmid library was subjected to Cas9 digestion, either in vivo or in vitro, and the resulting barcode information was obtained from transgenic embryos through Illumina NGS. Subsequently, an analysis was conducted on the data to evaluate barcode recombination (HR activity), estimate the average transgene copy number (CN), and assess end truncations and mutations at transgene-transgene junction sequences (MMEJ/NHEJ activity).

## Materials and methods

Animal ethics statement. The animal housing was implemented with the assistance of the Center for Laboratory Animal Genetic Resources at the Institute of Cytology and Genetics SB RAS, with support from the Ministry of Science and Higher Education of the Russian Federation (unique identifier of the project: RFMEFI62119X0023). The animals were maintained in a standard environment, with a temperature of 24 °C, relative air humidity of 40–50 %, and a 14-hour light/10-hour dark light cycle. The animals had access to food and water at all times. At the conclusion of the experiments, the remaining animals were euthanized via CO2 asphyxiation. All procedures and technical manipulations involving animals were conducted in accordance with the European Communities Council Directive of November 24, 1986 (86/609/EEC) and approved by the Bioethical Committee at the Institute of Cytology and Genetics SB RAS (Permission No. 45 from November 16, 2018).

DNA cloning. The pCAGGS-mCherry plasmid was a gift from Phil Sharp (Gurtan et al., 2012) (Addgene plasmid #41583). This vector was utilized for the insertion of paired barcodes. The barcoded plasmid library was constructed by assembling a linearized backbone, two barcoded oligonucleotides, and an AAVS1 PCR fragment using the NEBuilder® HiFi DNA Assembly mix (NEB). The sequences of the left and right barcodes were as follows: NNCGANNACTNNAT GNNACGNNCTGNNTCANN (left) and NNCTCNNGAA NNCGTNNCTANNTCGNNGTANN (right).

To synthesize gRNA against the AAVS1 site, a PCR fragment was amplified from the gRNA_AAVS1-T2 plasmid using a primer with a T7 overhang and treated with the MEGAscript ™ T7 Transcription Kit (Thermo Fisher, USA). The gRNA_AAVS1-T2 plasmid was a gift from George Church (Addgene plasmid #41818) (Mali et al., 2013). The resulting gRNA was purified using the RNA Clean &Concentrator™ Kit (Zymo Research, USA).

To assess the in vitro activity of Cas9, 1 μg of a selected clone from the plasmid library was incubated with 50–250 nM of the Cas9 protein (Biolabmix, Russia) and an equimolar amount of gRNA in an incubation buffer (20 mM HEPES pH 7.5, 125 mM KCl, 1 mM EDTA, 1 mM DTT, 6 mM MgCl2, 7 % glycerol). The reaction was incubated at 37 °C for 30 minutes, heat-inactivated at 65 °C, and visualized on a 1 % agarose gel.

Animals. Four-week-old female F1 C57BL/6J × DBA2/J (B6D2F1) mice were utilized for superovulation and oocyte collection due to their high hybrid vigor, while three-monthold male C57BL/6J mice were employed for sperm collection. Two-month-old pseudopregnant female CD-1 mice were utilized for embryo transfer into the oviducts, with isoflurane anesthesia. In vitro fertilization and embryo collection were conducted in accordance with the established protocols (Takeo et al., 2008; Takeo, Nakagata, 2010, 2011).

Pronuclear microinjection. Two distinct versions of the DNA injection mixture were prepared and delivered into the pronuclei. The RNP+ mix consisted of 1.65 μM Cas9 (Alt- R® S.p. Cas9 Nuclease V3, IDT, USA), 1.65 μM gRNA against AAVS1, and 8 ng/μl plasmid library diluted in TE buffer (0.01 M Tris–HCl, 0.25 mM EDTA, pH 7.4) (IDT). For the RNP-negative mix (RNP–), plasmid DNA was digested with the same RNP in vitro for one hour, gel-purified to remove Cas9, and diluted to 8 ng/μl with TE buffer. A quantity of DNA equivalent to one picoliter, comprising approximately 1,400 copies, was injected into the male pronuclei of in vitro fertilized zygotes. Subsequently, the microinjected zygotes were cultured in droplets of mHTF medium covered with mineral oil at 37 °C under 5 % CO2 in air for approximately 20 hours until the two-cell embryo stage was reached. Two-cell stage embryos were transferred into the oviducts of pseudopregnant CD-1 females at 0.5 days post-coitum. Genomic DNA from 13–14-day embryos was subjected to conventional PCR with Q5 polymerase (NEB, USA) and a set of primers spanning the region around transgene-transgene junctions (Tables S1–S2)1.


Supplementary Materials are available in the online version of the paper: https://vavilovj-icg.ru/download/pict-2025-29/appx1.pdf


Illumina sequencing and data analysis. Two distinct types of PCR products were amplified from the genomes of embryos #31, #34, #35, and #37. To prepare the inverse PCR NGS library, genomic DNA from transgenic embryos was digested overnight with KpnI-HF (NEB), which cuts twice within the transgene-transgene junction. The digested DNA was purified using AMPure XP magnetic beads (Beckman Coulter, USA), and 300 ng of the digested DNA was ligated overnight at 16 °C in a large reaction volume (100 μl) to facilitate self-ligation of transgene copies. The terminal barcodes of the self-ligated DNA fragments were amplified via PCR using Q5 polymerase and primers that spanned the barcode pairs (Table S2). The following PCR program was employed: the temperature was set to 98 °C for 30 seconds, followed by 35 cycles of 98 °C for 15 seconds, 62 °C for 30 seconds, and 72 °C for 30 seconds, with a final extension at 72 °C for 2 minutes. To remove any remaining PCR fragments resulting from undigested DNA, those of an appropriate size (216 bp) were excised from the agarose gel.

To prepare the PCR library corresponding to transgenetransgene junctions, the internal junctions were amplified with primers, one of which was in close proximity to the junction and the other of which was situated adjacent to the barcode (Tables S1 and S2). The PCR products were generated for both orientations and purified using AMPure XP magnetic beads. The PCR products were prepared with the KAPA HyperPrep Kit (Roche, Switzerland) using two amplification cycles, pooled together, and sequenced on the Illumina HiSeq 2500 platform (Illumina, USA). The quality of the libraries was evaluated using an Agilent 2100 Bioanalyzer (Agilent, USA) and the Qubit dsDNA HS assay Kit (Life Technologies, USA).

The processing of NGS data (Table S3) was conducted in accordance with the methodology described in our previous report (Smirnov et al., 2020) with minor modifications. The analysis of NGS data comprises the following steps. Initially, the data were demultiplexed according to the samples. Metrics based on the Levenshtein distance were employed to identify primer sequences at the 3ʹ and 5ʹ ends of each read pair. Subsequently, barcodes were sought within read pairs exhibiting regular patterns (i. e., head barcode NNCGANN ACTNNATGNNACGNNCTGNNTCANN and tail barcode NNCTCNNGAANNCGTNNCTANNTCGNNGTANN). Furthermore, the number and percentage of read pairs sharing identical barcodes and identical barcode pairs were calculated. The resulting set of barcode pairs was subjected to a filtration process to produce the final set of pairs. Finally, the barcode pairs were visualized using the Network module of the vis.js framework (http://visjs.org/). All of the aforementioned computations were conducted on a high-throughput computational cluster at Novosibirsk State University. The following Python modules were utilized: the Biopython v1.79 module (Cock et al., 2009, 2010) was utilized. Next-generation sequencing data processing (re v2.2.1) utilizes regular expressions (Levenshtein v0.12.1). The Levenshtein distance was calculated using the Pandas v1.3.3 module for processing table data, while the NetworkX 2.6.2 module was employed to determine the number of pairs.

Droplet digital PCR. The Droplet Digital PCR (ddPCR) method was employed to ascertain the transgene copy number in the embryos. The procedure was conducted using ddPCR Supermix for Probes (no dUTP) and QX100 ddPCR Systems (Bio-Rad, USA), in accordance with the instructions provided by the manufacturer. First, 900 ng of genomic DNA was digested overnight with 20 U of BamHI-HF (NEB) in CutSmart buffer. One microliter of genomic DNA (30 ng) was added to the ddPCR mix (1× ddPCR Supermix, 900 nM primers, 250 nM probes), and the reactions were carried out according to the following program: the reaction was initiated at 95 °C for 10 minutes, followed by 41 cycles of 94 °C for 10 seconds and 61 °C for 1 minute. The final step was conducted at 98 °C for 7 minutes and 20 °C for 30 minutes. All the steps were conducted with a ramp rate of 2 °C/s. ddPCR was performed in two independent technical replicates. The sequences of the primers and probes are provided in Table S4. The data were subsequently analyzed using QuantaSoft (Bio-Rad). The threshold for both genes was set at 5,000.

## Results

Generation of transgenic embryos by pronuclear microinjection

**Fig. 1.**
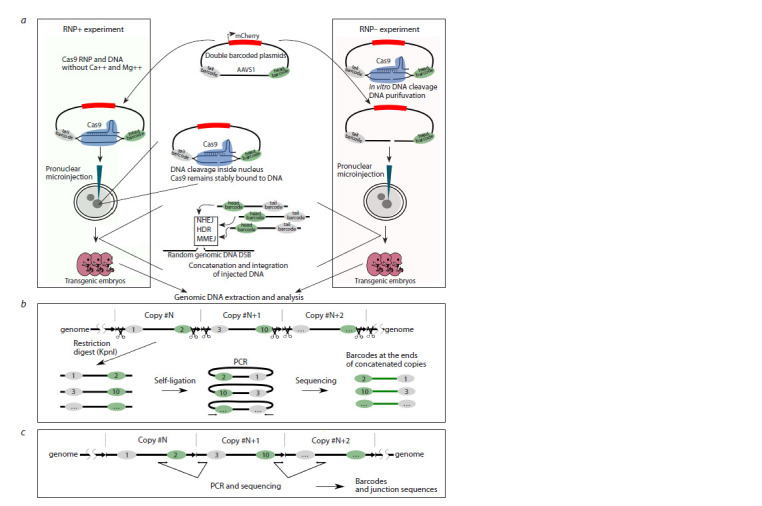
Outline of the experimental approach. a – the barcoded plasmid library was prepared for microinjections. A plasmid library comprising a pair of 32 bp barcodes separated by the human AAVS1 fragment was employed to prepare two experimental setups. In the RNP+ experiments, Cas9 and gRNA targeting AAVS1 were co-injected with the plasmid library into pronuclei. In vivo, plasmid linearization occurs within the pronucleus. The right-hand illustration depicts the second experimental setup. The plasmid library is digested with RNP in vitro, purified via gel electrophoresis, and injected as a purified, linear DNA construct (RNP–). Following the injection, the linear molecules undergo recombination, forming either concatemers or single copies, which are then detected through PCR genotyping; b – the barcodes present in the genomic DNA are subjected to analysis through the process of inverse PCR. The concatenated copies are then separated by KpnI (scissors) and re-ligated. PCR products from the newly formed junctions are then subjected to analysis by NGS; c – internal junctions between copies are subjected to PCR amplification and subsequent sequencing.

**Fig. 2.**
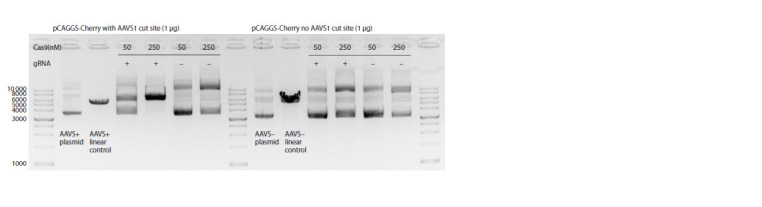
Preparation of barcoded transgenes for microinjection. In vitro cleavage of barcoded plasmid using Cas9 RNP. The image on the left depicts a barcoded clone with an AAVS1 site, while the image on the right illustrates the original backbone without an AAVS1 site. The incubations were maintained at 37 °C for a period of 30 minutes.

For the experiments, we used a barcoded plasmid library, which was based on the pCAGGS-mCherry vector (Fig. 1a, middle section). In brief, the vector contains a pair of 32-base pair barcodes (14 random positions) separated by filler DNA. In the present experiments, a fragment of the human AAVS1 site was utilized as filler DNA due to its well-characterized gRNA sites (Chr19:55115511-55115987, 477 bp) (Mali et al., 2013; Maggio et al., 2014). One of the gRNA sites (5ʹ-GGGGCCACTAGGGACAGGAT-3ʹ) was employed as a Cas9 target to linearize plasmid DNA and generate transgenes with barcoded ends (Fig. 1a). In vitro tests with the Cas9 protein demonstrated that the synthesized gRNA exhibited robust performance against the plasmid (Fig. 2). To analyze the plasmid library, Illumina paired-end sequencing of the barcodes was performed. The plasmid library was found to consist of 102,685 unique barcode pairs. In a typical pronuclear microinjection experiment, approximately one thousand molecules are introduced into the pronucleus. The diversity of the library, comprising approximately 100,000 unique sequences, allows for the injection of a vast number of molecules into a single pronucleus, with minimal likelihood of identical sequences being present

To understand how the presence of Cas9 at the ends of molecules affects the DSB repair mechanisms involved in concatenation and integration of exogenous DNA, we conducted two experiments on DNA microinjection into pronuclei (Fig. 1a). In one variant, the original circular plasmid library was combined with the Cas9 protein and gRNA, which formed a ribonuclear protein complex (RNP) in standard TE buffer (Fig. 1a, left section, “RNP+”). The absence of Mg2+ ions in the buffer precludes Cas9 endonuclease activity prior to pronuclear microinjection. The concentration of Cas9/gRNA RNP was increased to 1.65 μM, which is above the concentration typically used, in order to ensure robust plasmid linearization. In the second experiment, the DNA was subjected to in vitro digestion using Cas9 (Fig. 1a, right section). The linearized DNA was purified via gel electrophoresis and subsequently used for microinjection (henceforth referred to as “RNP–”). In both experiments, approximately 1,400 copies were injected per zygote.

**Fig. 3.**
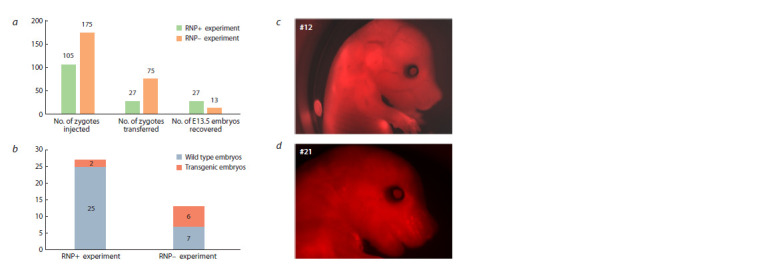
Screening of the transgenic embryos a – statistical analysis of pronuclear microinjection experiments; b – PCR analysis of E13–14 embryos; c–d – visualization of mCherry fluorescence through microscopic examination.

Genomic DNA was extracted from the embryos at the 13– 14-day (E13–14) developmental stage. A total of 27 embryos were collected from the RNP+ experiment, and 13 embryos were collected from the RNP– experiment (Fig. 3a). PCR genotyping with primers for the mCherry gene and transgene- transgene junctions revealed the presence of seFig veral positive embryos, with 2/27 for RNP+ and 6/13 for RNP– (Fig. 3b). Embryos derived from the RNP– experiment exhibited positive results for transgene-transgene junctions, a hallmark of concatemers (Fig. S1). Of the transgenic embryos, only two exhibited mCherry fluorescence (Fig. 3c–d).

To ascertain the transgene copy number, droplet digital PCR was conducted using a probe directed against the mCherry region within the transgene (Fig. S2). Embryos from the RNP+ experiment exhibited low CN (0.07, 0.37), whereas embryos from the RNP– experiment demonstrated a higher number of copies on average (CN = 0.06, 0.89, 1.87, 2.8, 5.84, 6.38) (Fig. S2) (see realistic estimates below). It should be noted that in relation to ddPCR, CN < 1 or partial CN values indicate tissue mosaicism, whereby the transgene exists only in a portion of the embryo cells. This could reflect delayed DNA integration.

Barcode analysis

**Fig. 4.**
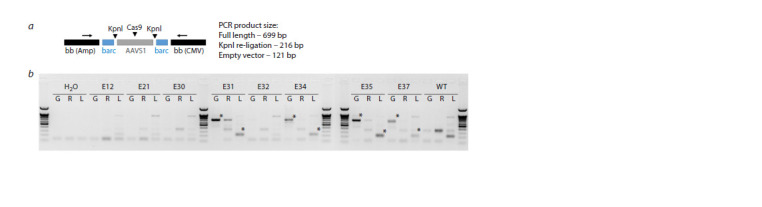
Generation of PCR fragments for the barcode NGS analysis using inverse PCR. a – the positions of the PCR primers (indicated by arrows) and the KpnI/Cas9 cut sites at the hypothetical transgene-transgene junction are shown. The empty vector is the original pCAGGS backbone devoid of the AAVS1 element; b – generation of PCR products for NGS. G: Untreated genomic DNA; R: Genomic DNA treated with KpnI; L: Genomic DNA after re-ligation for inverse PCR. Inverse PCR entails the digestion of genomic DNA with KpnI and subsequent re-ligation to create self-ligated ends. Consequently, the size of the PCR product is diminished from 699 base pairs to 216 base pairs. The intensity of the bands is proportional to the copy number. Legend: H2O was used as the negative control, E12–E37 were the transgenic embryos, and WT was the wild-type control. The PCR products selected for NGS analysis are indicated by asterisks. The DNA length marker is 100 base pairs in length.

Barcodes derived from concatemers in embryos were sequenced using Illumina paired-end reads. Two types of PCR products were prepared for barcode sequencing, following the methodology described in our previous report (Fig. 1b–c) (Smirnov et al., 2020). In summary, barcode pairs at the ends of concatenated transgenes were amplified via PCR through self-molecular ligation (Fig. 1b). This method facilitates the excision and self-ligation of transgene molecules, which enables the reading of terminal barcodes in the copies. The KpnI sites utilized for the fragmentation of concatemers in inverse PCR are situated between barcodes and yield 216 bp PCR fragments subsequent to ligation (Fig. 4a). Additionally, internal junctions, defined as nucleotide sequences situated between the copies, were subjected to sequencing (Fig. 1c). In total, barcode data were obtained for four multicopy RNP embryos (via inverse PCR and junction PCR) and two single-copy RNP+ embryos (due to the lack of junctions, only inverse PCR was conducted). To investigate recombination of the barcode sequences, a comparison was made between the barcodes from the transgenic embryos and the list of barcodes from the original plasmid library.

**Fig. 5.**
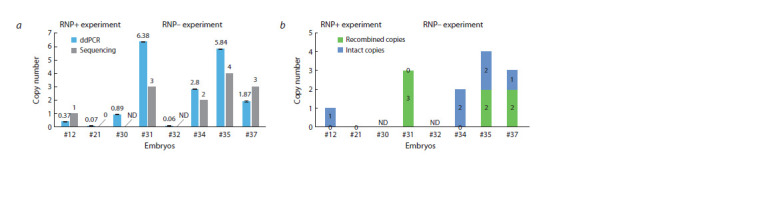
A comparison of CN measurements derived from ddPCR and NGS barcode analysis. a – CN estimates derived from ddPCR and NGS in transgenic embryos (#12–37). ND – no data available. A CN value of less than 1 is indicative of tissue mosaicism; b – estimation of the proportion of copies with barcode recombination (green).

First, we employed NGS barcode data to ascertain the actual CN in the embryos. It is established that the ddPCR results typically exhibit an understated CN due to the mosaic nature of transgenic embryos. By employing barcodes as unique copy identifiers, we were able to calculate the actual CN (Fig. 5). The CN data exhibited a strong correlation with the ddPCR data, with a slight tendency towards an increase in CN. Embryos with a very low CN (0.06–0.89) as estimated by ddPCR, such as those designated #12/#21 from the RNP+ experiment or #30/#32 from the RNP– experiment, exhibited no transgene-transgene junctions, indicating that they possess a single or truncated copy. It is noteworthy that the truncation of transgene ends during concatenation has the potential to delete the site of a barcode and thereby obscure the calculation of a copy. Nevertheless, in our particular case, an examination of the PCR junctions did not reveal any evidence of terminal truncations, in addition to the presence of a typical transgenetransgene junction (Fig. S1b).

**Fig. 6.**
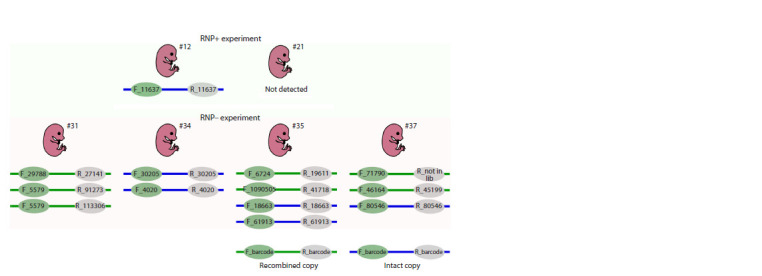
The barcodes observed in four multicopy RNP– embryos (#31, #34, #35, #37) and one RNP+ embryo (#12). Barcoded transgenes are represented as colored lines with barcode numbers at the ends. Intact transgene copies are indicated in blue (representing the original barcode pairs from the library), while copies with recombined barcodes are shown in green. The top panel depicts embryos from the RNP+ experiment. The bottom panel depicts four RNP– embryos with their corresponding transgene copies. “R_not in lib” indicates that the barcode was not identified within the plasmid library.

**Fig. 7.**
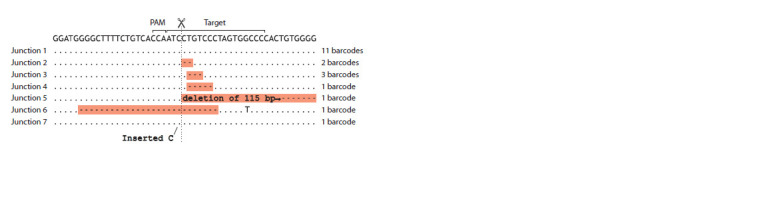
Nucleotide sequences at the transgene-transgene junctions.

As anticipated, a substantial proportion (53 %) of barcode pairs in concatemers were observed to have undergone shuffling (Fig. 5b and 6). These barcode pairs were not initially present in the plasmid library and appear to have arisen from extrachromosomal end recombination between transgene copies (Smirnov et al., 2020). One barcode was observed to be linked with two distinct partners (Fig. 6, Embryo #31, F_5579), indicating that a single donor molecule was replicated on at least two occasions during the recombination process (Smirnov et al., 2020). This evidence confirms that transgene copies prepared by in vitro Cas9 cleavage engage in HR pathways during concatenation, in contrast to the copies from the RNP+ experiment. An internal junction analysis revealed that 11 out of the 20 junctions attributed to specific barcodes did not contain any mutations (Fig. 7). The mutated junction sequences were found to comprise a variety of deletions. Two to five base pair deletions, a large 115 base pair end truncation, and a two-sided 27 base pair deletion (Fig. 7). It should be noted that the DNA ends in question were produced by Cas9 cleavage in vitro and were likely trimmed by Cas9 during the incubation period, prior to interaction with the DNA repair machinery.

## Discussion

The improvement of efficiency in Cas9-assisted transgenesis will have a profound impact on experiments with a low baseline success rate, such as those involving > 2 kb donor integration for humanization or genome modifications of farm animals with long reproductive cycles and a lack of scaling. The ability to leverage Cas9 activity hinges on a comprehensive understanding of its interactions with cellular DNA repair pathways. A number of studies have demonstrated that Cas9 forms an unusually stable nuclease-substrate complex and remains bound to the DSB it generates (Jinek et al., 2014; Nishimasu et al., 2014; Stephenson et al., 2018). The precise manner by which cellular repair systems recognize a DSB and the extent to which the Cas9 protein at the DSB influences the choice of repair pathway remain largely unknown. It has been demonstrated that the Cas9 complex can be dislodged from a double-stranded break by RNA polymerase, but only if the single-guide RNA (sgRNA) of Cas9 is annealed to the DNA strand that is used as the template by the RNA polymerase (Clarke et al., 2018).

New evidence indicates that Cas9 should be removed by HLTF to initiate end processing (Reginato et al., 2024), as post-cleavage Cas9 complexes have been shown to impede DNA from MRE11 (Maltseva et al., 2023; Reginato et al., 2024). Concatenation reporter used in the present study has some unique properties. It could be applied in pronuclear microinjection and allows to inspect many end-joining events at once. We utilized the reporter in combination with Cas9 to study three parameters: transgene CN (DNA end accessibility), barcode recombination (DNA end participation in HR), and internal junctions (NHEJ/MMEJ DNA end processing).

The evaluation of the transgene CN by ddPCR and NGS indicated the absence of concatemers in the RNP+ experiments (E12, E21). Two potential explanations for the absence of concatemers in RNP+ can be postulated. Firstly, it is possible that Cas9 may block cleaved ends from processing by DNA repair factors, thereby preventing end resection and concatenation, which represents the primary mechanism of CN increase (Smirnov et al., 2020). Secondly, it may be that Cas9 is less active in cutting plasmid libraries, which would result in a reduction in the number of linear copies that integrate rarely and/or late in the zygote division. However, the RNP concentration used for pronuclear microinjection was higher than the average recommended by D.W. Harms et al. (2014). This concentration should have been sufficient to linearize the library (1400 DNA copies), as most genomeediting experiments using a lesser amount of RNP generally show high rates of genomic modifications.

Given the overall low copy number of the generated concatemers, the barcode recombination assay was not particularly informative. It is noteworthy that some embryos that tested positive by PCR or mCherry fluorescence did not produce barcodes from the NGS analysis. This indicates that the integrated copy/copies are damaged. While the mCherry cassette is located in the middle of the transgene, the ends of the transgenes are more susceptible to truncations that result in the loss of one or both barcodes. Barcode switching was detected (Fig. 6) and a barcode copying event, which also serves as an indicator of HR activity, was observed. The result of barcode switching (53 %) was not significantly different from that of the previous report (80 % recombined copies) (Smirnov et al., 2020). In conclusion, the presented evidence suggests that transgene copies prepared by in vitro Cas9 cleavage may engage in HR pathways during concatenation, in contrast to the copies from the RNP+ experiment.

The analysis of internal junctions revealed a typical assortment of Cas9-generated deletions, with occasional instances of nucleotide insertions (Schimmel et al., 2017). It is established that Cas9 produces heterogeneous ends due to endonucleolytic degradation of the DNA by endonuclease domains, with higher activity towards the PAM distal fragment (Stephenson et al., 2018). This may explain the observed asymmetry in deletions at the junctions (Fig. 7, with the PAM distal fragment to the right). It is noteworthy that intact junctions were observed with considerable frequency among the variants. It was not possible to ascertain with certainty whether the junctions originated from undigested vector or de novo ligation of blunt DNA ends. However, given that in vitro digested DNA was gel-purified (Fig. 2, linear fragment) and should not contain a significant proportion of undigested circular form, it was assumed that the latter was the case. These junction signatures differ from those typically generated by restriction enzymes in standard pronuclear microinjection experiments (Rohan et al., 1990; Dai et al., 2010). For example, in our previous study, we selected the BsmBI enzyme to linearize the barcoded vector and create 4 nt incompatible protruding 5ʹ-ends. Following concatenation, transgene molecules exhibited a loss of approximately 5–10 nucleotides at the junction sites, with no discernible asymmetry (Smirnov et al., 2020).

In comparison to the barcoded library previously described (Smirnov et al., 2020), the pCAGGS-Cherry-based reporter exhibited a tenfold increase in barcode diversity and an extended barcode length (32 bp vs 17 bp). This allows for unambiguous interpretation of complex barcode recombination patterns, including the copying of a single barcode to multiple copies (Fig. 6). Nevertheless, in theory, the relatively low overall CN compared to other experiments (Smirnov et al., 2020) may indicate that the linear copies are impeded in their end recombination. In this instance, the absence of concatemers may be attributed to the augmented barcode length, which impedes homology search and head-to-tail end joining.

## Conclusion

In conclusion, our findings demonstrate that the DNA ends generated by Cas9 in vivo undergo a distinct processing pathway compared to the “clean” ends. The microinjection of the barcoded library in combination with CRISPR endonucleases represents a fruitful assay that can be augmented further. This could be achieved, for example, by chemical modifications of the DNA ends or by co-injection of the NHEJ/HR factors. It would be beneficial to gain a deeper understanding of how Cas9 affects end recombination during concatenation, with the aim of preventing unwanted concatenation of donor molecules or stimulating end processing at the genomic site in the future.

## Conflict of interest

The authors declare no conflict of interest.

## References

Abe T., Inoue K., Furuta Y., Kiyonari H. Pronuclear microinjection
during S-phase increases the efficiency of CRISPR-Cas9-assisted
knockin of large DNA donors in mouse zygotes. Cell Rep. 2020;
31(7):107653. doi 10.1016/j.celrep.2020.107653

Clarke R., Heler R., MacDougall M.S., Yeo N.C., Chavez A., Regan
M., Hanakahi L., Church G.M., Marraffini L.A., Merrill B.J.
Enhanced bacterial immunity and mammalian genome editing via RNA-polymerase-mediated dislodging of Cas9 from double-strand
DNA breaks. Mol Cell. 2018;71(1):42-55.e8. doi 10.1016/j.molcel.
2018.06.005

Cock P.J.A., Antao T., Chang J.T., Chapman B.A., Cox C.J., Dalke A.,
Friedberg I., Hamelryck T., Kauff F., Wilczynski B., De Hoon M.J.L.
Biopython: freely available Python tools for computational molecular
biology and bioinformatics. Bioinformatics. 2009;25(11):1422-
1423. doi 10.1093/bioinformatics/btp163

Cock P.J.A., Fields C.J., Goto N., Heuer M.L., Rice P.M. The Sanger
FASTQ file format for sequences with quality scores, and the Solexa/
Illumina FASTQ variants. Nucleic Acids Res. 2010;38(6):
1767-1771. doi 10.1093/nar/gkp1137

Dai J., Cui X., Zhu Z., Hu W. Non-homologous end joining plays a
key role in transgene concatemer formation in transgenic zebrafish
embryos. Int J Biol Sci. 2010;6(7):756-768. doi 10.7150/ijbs.6.756

Danner E., Lebedin M., De La Rosa K., Kühn R. A homology independent
sequence replacement strategy in human cells using a
CRISPR nuclease. Open Biol. 2021;11(1):200283. doi 10.1098/rsob.
200283

Gurtan A.M., Lu V., Bhutkar A., Sharp P.A. In vivo structure-function
analysis of human Dicer reveals directional processing of precursor
miRNAs. RNA. 2012;18(6):1116-1122. doi 10.1261/rna.032680.112

Harms D.W., Quadros R.M., Seruggia D., Ohtsuka M., Takahashi G.,
Montoliu L., Gurumurthy C.B. Mouse genome editing using the
CRISPR/Cas system. Curr Protoc Hum Genet. 2014;83:15.7.1-
15.7.27. doi 10.1002/0471142905.hg1507s83

Jinek M., Jiang F., Taylor D.W., Sternberg S.H., Kaya E., Ma E., Anders
C., Hauer M., Zhou K., Lin S., Kaplan M., Iavarone A.T.,
Charpentier E., Nogales E., Doudna J.A. Structures of Cas9 endonucleases
reveal RNA-mediated conformational activation. Science.
2014;343(6176):1247997. doi 10.1126/science.1247997

Maggio I., Holkers M., Liu J., Janssen J.M., Chen X., Gonçalves
M.A.F.V. Adenoviral vector delivery of RNA-guided CRISPR/
Cas9 nuclease complexes induces targeted mutagenesis in a diverse
array of human cells. Sci Rep. 2014;4(1):5105. doi 10.1038/
srep05105

Mali P., Yang L., Esvelt K.M., Aach J., Guell M., DiCarlo J.E., Norville
J.E., Church G.M. RNA-guided human genome engineering
via Cas9. Science. 2013;339(6121):823-826. doi 10.1126/science.
1232033

Maltseva E.A., Vasil’eva I.A., Moor N.A., Kim D.V., Dyrkheeva N.S.,
Kutuzov M.M., Vokhtantsev I.P., Kulishova L.M., Zharkov D.O.,
Lavrik O.I. Cas9 is mostly orthogonal to human systems of DNA
break sensing and repair. PLoS One. 2023;18(11):e0294683. doi
10.1371/journal.pone.0294683

Nishimasu H., Ran F.A., Hsu P.D., Konermann S., Shehata S.I., Dohmae
N., Ishitani R., Zhang F., Nureki O. Crystal structure of Cas9
in complex with guide RNA and target DNA. Cell. 2014;156(5):
935-949. doi 10.1016/j.cell.2014.02.001

Reginato G., Dello Stritto M.R., Wang Y., Hao J., Pavani R., Schmitz M.,
Halder S., Morin V., Cannavo E., Ceppi I., Braunshier S., Acharya
A., Ropars V., Charbonnier J.-B., Jinek M., Nussenzweig A.,
Ha T., Cejka P. HLTF disrupts Cas9-DNA post-cleavage complexes
to allow DNA break processing. Nat Commun. 2024;15(1):5789. doi
10.1038/s41467-024-50080-y

Richardson C.D., Ray G.J., DeWitt M.A., Curie G.L., Corn J.E. Enhancing
homology-directed genome editing by catalytically active
and inactive CRISPR-Cas9 using asymmetric donor DNA. Nat Biotechnol.
2016;34(3):339-344. doi 10.1038/nbt.3481

Rohan R.M., King D., Frels W.I. Direct sequencing of PCR-amplified
junction fragments from tandemly repeated transgenes. Nucleic
Acids
Res. 1990;18(20):6089-6095. doi 10.1093/nar/18.20.6089

Sakuma T., Nakade S., Sakane Y., Suzuki K.-I.T., Yamamoto T. MMEJassisted
gene knock-in using TALENs and CRISPR-Cas9 with the
PITCh systems. Nat Protoc. 2016;11(1):118-133. doi 10.1038/nprot.
2015.140

Schimmel J., Kool H., Van Schendel R., Tijsterman M. Mutational
signatures of non‐homologous and polymerase theta‐mediated endjoining
in embryonic stem cells. EMBO J. 2017;36(24):3634-3649.
doi 10.15252/embj.201796948

Smirnov A., Fishman V., Yunusova A., Korablev A., Serova I., Skryabin
B.V., Rozhdestvensky T.S., Battulin N. DNA barcoding reveals
that injected transgenes are predominantly processed by homologous
recombination in mouse zygote. Nucleic Acids Res. 2020;
48(2):719-735. doi 10.1093/nar/gkz1085

Stephenson A.A., Raper A.T., Suo Z. Bidirectional degradation of DNA
cleavage products catalyzed by CRISPR/Cas9. J Am Chem Soc.
2018;140(10):3743-3750. doi 10.1021/jacs.7b13050

Suzuki K., Tsunekawa Y., Hernandez-Benitez R., Wu J., Zhu J.,
Kim E.J., Hatanaka F., Yamamoto M., Araoka T., Li Z., Kurita M.,
Hishida T., Li M., Aizawa E., Guo S., Chen S., Goebl A., Soligalla
R.D., Qu J., Jiang T., Fu X., Jafari M., Esteban C.R., Berggren
W.T., Lajara J., Nuñez-Delicado E., Guillen P., Campistol J.M.,
Matsuzaki F., Liu G.-H., Magistretti P., Zhang K., Callaway E.M.,
Zhang K., Belmonte J.C.I. In vivo genome editing via CRISPR/Cas9
mediated homology-independent targeted integration. Nature. 2016;
540(7631):144-149. doi 10.1038/nature20565

Taheri-Ghahfarokhi A., Taylor B.J.M., Nitsch R., Lundin A., Cavallo
A.-L., Madeyski-Bengtson K., Karlsson F., Clausen M., Hicks R.,
Mayr L.M., Bohlooly-Y.M., Maresca M. Decoding non-random mutational
signatures at Cas9 targeted sites. Nucleic Acids Res. 2018;
46(16):8417-8434. doi 10.1093/nar/gky653

Takeo T., Nakagata N. Combination medium of cryoprotective agents
containing l-glutamine and methyl-β-cyclodextrin in a preincubation
medium yields a high fertilization rate for cryopreserved
C57BL/6J mouse sperm. Lab Anim. 2010;44(2):132-137. doi
10.1258/la.2009.009074

Takeo T., Nakagata N. Reduced glutathione enhances fertility of frozen/
thawed C57BL/6 mouse sperm after exposure to methyl-betacyclodextrin.
Biol Reprod. 2011;85(5):1066-1072. doi 10.1095/
biolreprod.111.092536

Takeo T., Hoshii T., Kondo Y., Toyodome H., Arima H., Yamamura K.,
Irie T., Nakagata N. Methyl-beta-cyclodextrin improves fertilizing
ability of C57BL/6 mouse sperm after freezing and thawing by facilitating
cholesterol efflux from the cells. Biol Reprod. 2008;78(3):
546-551. doi 10.1095/biolreprod.107.065359

